# Mycotic Aortic Aneurysm, a very sudden development for a preexisting Aortic Aneurysm: Case report

**DOI:** 10.1016/j.ijscr.2024.110034

**Published:** 2024-07-13

**Authors:** George Bashour, Houssam Kinjo, Hussein Kaada, Rana Issa

**Affiliations:** a Faculty of Medicine, Tishreen University, Latakia, Syria; b Department of Vascular Surgery, Tishreen University Hospital, Latakia, Syria; c Department of Thoracic Surgery, Tishreen University Hospital, Latakia, Syria; d Department of Pathology, Tishreen University Hospital, Latakia, Syria

**Keywords:** Mycotic Aortic Aneurysm, Vascular surgery, Infectious aortitis

## Abstract

**Introduction:**

An Infectious Aortic Aneurysm (IAA), also known as a Mycotic Aortic Aneurysm (MAA), is a rare lesion of the aorta resulting from an infection of its wall.

**Presentation:**

A male patient in his 70s presented to our emergency department with fever, intense abdominal pain, and a pulsatile mass in the middle of the abdomen. A Computed Tomography (CT) angiography scan was done two weeks earlier and it showed a 6.6 cm subrenal aortic aneurysm. A new CT angiography scan revealed a 3.4 cm growth (10*10*9.3) with periaortic fluids. The diagnosis of MAA was considered, and emergency surgery was performed.

**Discussion:**

MAA is a rare disease characterized by a high risk of rupture and a high mortality rate, up to 43 %, despite the advances in treatment techniques.

Adjacent infection is a rare cause for MAA especially on a preexisting aneurysm.

The two main surgical approaches are Open Surgical Repair (OSR) and Endo-Vascular Repair (EVR). We opted for OSR with careful debridement because EVR was not available at our center and the huge size of the aneurysm posed high rupture risk.

**Conclusion:**

This case demonstrates the importance of close monitoring and early intervention for aneurysms, particularly in cases with adjacent infection. Moreover, the rapid growth rate and rupture risk demands more urgent intervention if the MAA is suspected.

## Highlights

• Mycotic aortic aneurysm is a lesion resulting from infection of the aorta wall.
• The growth rate of MAA has not been discussed in the literature.
• This case demonstrates a 3.4 cm growth of the MAA in 2 weeks period.

## 1 Introduction

A Mycotic Aortic Aneurysm (MAA), also known as an Infectious Aortic Aneurysm (IAA), is a lesion of the aorta resulting from an infection of its wall [1].

This condition was firstly described by Osler in 1851 in an autopsy of a young man in the context of malignant endocarditis and he coined the term “Mycotic Aortic Aneurysm” [2]. MAAs represent approximately 1.5 % of aortic aneurysms and can affect any aortic portion, though the predominant location remains intra-abdominal [3,4]. This type of aneurysm is characterized by a rapid growth, leading to a high risk of rupture if left untreated. The exact pathogenesis is still unknown [5,6].

There are two main surgical techniques: Endo-Vascular Repair (EVR) and Open Surgical Repair (OSR). EVRs are preferred as they have more favorable results. However, there are currently no recommendations from scientific societies regarding the surgical strategy for managing AAI due to the lack of evidence [3,7].

Here we present a sporadic case of MAA developed from a preexisting aortic aneurysm due to infectious gastroenteritis and rapidly grew without rupturing. We also describe the technique used in treatment in our low-resource settings. This work has been reported in line with the SCARE criteria [8].

## 2 Presentation

A male patient in his 70s, known for high blood pressure and COPD, was admitted to our emergency department for intense lower back and abdominal pain. A pulsatile mass could be palpated in the middle of the abdomen. Temperature 39.5 °C, heart rate 130/min, BP 180/100 mmHg. The left renal compartment was sensitive to percussion. The recent lab results showed Leukocytes 22,400 WBC/mm^3^, including 85 % neutrophils, hemoglobin 11 mg/dl, creatinine 162 μmo/l, and CRP at 220 mg/l.

The patient mentioned that he went to a gastrointestinal clinic 20 days ago for moderate abdominal pain with fever, nausea, and diarrhea. Previous labs showed Leukocytosis (12,500 WBC/mm^3^ with 80 % neutrophils), anemia (10 mg/dl), moderately elevated creatinine (130 μmol/l), and CRP (35 mg/l).

**Fig. 1:**
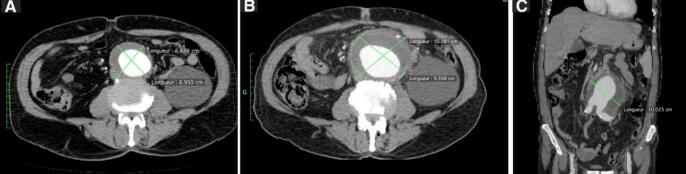
Computed tomography (CT) scan with contrast, axial section: demonstrates the aneurysm with no signs of inflammation (A), axial section:2 weeks later, a 3.4 cm growth with signs of periaortic inflammation (B) and a coronal section showing extension of the 10 cm mycotic aneurysm (C).

2 weeks prior to admission, a Computed Tomography (CT) scan with contrast (Fig. 1a) showed a subrenal aortic aneurysm with a diameter of 6.5 cm and without any sign of rupture or infection, left primary iliac aneurysm with a diameter of 4 cm, and moderate dilatation of the small bowel loop. Their initial diagnosis was gastroenteritis and they prescribed Augmentin (1 g po).

The second CT scan with contrast (Fig. 1b) showed a rapid increase in the diameter of the aortic aneurysm (10*10*9.3 cm), pre-cracked, and periaortic fluid accumulation. The iliac aneurysm remains stable. The patient was admitted to our vascular surgery department, and we mainly considered the diagnosis of infectious aortitis with a mycotic aneurysm. IV antibiotic therapy with Meropenem was initiated. Before the start of the treatment, blood samples were taken for culture and were negative.

**Fig. 2:**
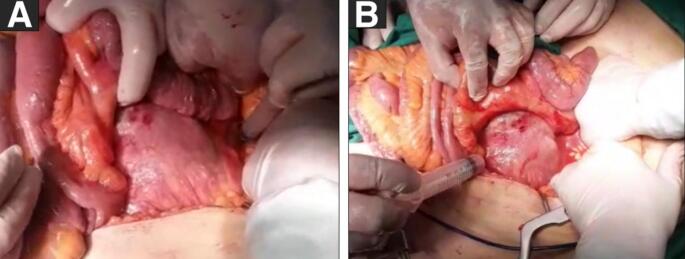
Surgical view: shows the mycotic aortic aneurysm being dissected and culture samples taken from the periaortic area (A&B).

Two days later, the decision was made to perform emergency surgery. (Fig. 2) Under general anesthesia, we gained access to the retroperitoneal MAA via a midline skin incision. After clamping the infrarenal aorta and both iliac arteries, we found significant pus in the perianeurysmal area and in the aneurysm wall. Subsequently, we performed thorough and careful local debridement, and resection of the perianeurysmal tissue and aneurysm sac, followed by extensive irrigation. A flattening is done for the left primary iliac aneurysm. We interposed the aorta-bifemoral artery with a bifurcated polytetrafluoroethylene (Dacron) graft. The region was washed with amikacin (500 mg distilled in 500 ml saline). The graft was covered with an omental flap.

**Fig. 3:**
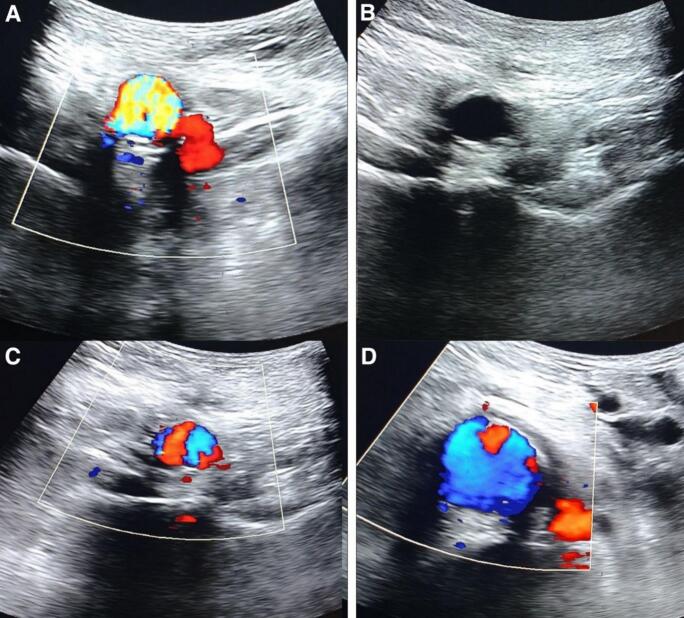
Follow-up Doppler ultrasound after 18 months demonstrates the patent graft with no complications (A&B&C&D).

We continued the antibiotic treatment with Meropenem for 7 days. The results of culture tests of samples taken from perianeurysmal area, thrombus, and aneurysm wall were negative. The patient showed relatively rapid improvement and was discharged after a 7-day follow-up and was put on oral ciprofloxacin and levofloxacin for 3 weeks. The infection has subsided, and the patient reported feeling well. The patient was followed-up for 18 months with Doppler ultrasound and it was normal (Fig. 3).

## 3 Discussion

MAA is a rare disease and life- threatening in the short term due to the rapid progression and the destruction of the aortic wall. In addition, it requires an appropriate medical and surgical intervention [1,2].

The growth rate of MAA has not been discussed in the literature except in some case reports [9,10]. It has been agreed that infection can cause a rapid growth of the aneurysm, as it leads to the destruction of the arterial wall, increasing the risk of its rupture. MAA rupture rate at first presentation can be as high as 44 % [3].

MAA etiology is still a point of discussion. The main proposed causes are septic embolism, adjacent infection, and direct blood vessel contamination during trauma or surgical interventions [5,6,11]. Adjacent infection is a rare cause for MAA especially in preexisting aneurysm.

MAA usually presents with fever 67 %, pain 77 %, and shock 15 % [3]. Clinical and laboratory findings are usually non-specific and may include pulsating mass, leukocytosis, and elevated sedimentation rate [12].

The diagnosis of MAA is mainly based on CT scans and clinical examination. The MAA features on CT include saccular outpouching, contrast enhancement, irregular arterial walls, and periaortic findings that include gas, which is rare but highly suggestive of MAA, soft tissue stranding, fluid, concentric inflammatory response, and mass formation [5,6]. Early diagnosis is very crucial for the treatment and positive outcomes due to the high rupture risk [5]. Constant monitoring is important to assess the growth rate and preventing serious complications of the aneurysm. Bacteremia is common and culture should be ordered although it can be negative. Negative cultures are associated with better outcomes [3,13].

In our case, the aneurysm was diagnosed coincidentally as it was an asymptomatic 6.5 cm aortic aneurysm. However, there was poor follow-up by the clinic and the patient was not transferred to any vascular specialist to be assessed. The aneurysm developed into MAA and the adjacent infection of the gastrointestinal tract was a suggested cause. Another probable mechanism is sepsis caused by the failed treatment of the infection, although we believe that the sepsis happened after the development of the MAA as the patient reported that the high temperature occurred on the previous day of admission. The Augmentin course that was prescribed by the clinic lasted for 2 weeks before the patient was admitted to our department. We started him on Meropenem due to the lack of response on Augmentin.

The rarity of MAA and the heterogeneity of the patients contribute to the lack of evidence and consensus on managing this disease. Only observational studies have been done so far with no standardized reporting [3].

Medical treatment alone, combining suspensive antibiotic therapy and blood pressure control, is often inadequate due to the persistence of the infection which systematically leads to short-term rupture. OSR consisting of total resection of the aneurysm associated with ablation of adjacent tissues and “in situ” reconstruction appears to be the appropriate treatment [3].

Despite optimal patient care, post-operative mortality of MAA is between 18 and 43 % [1,6,12]. EVR has been getting more attention in recent years with lower early mortality compared with OSR [7].

EVR was associated with a higher infection rate compared with OSR (5.7 % vs 0.9 %) [7].

In our case, we opted for OSR as an emergent option due to the extremely rapid growth and size of the aneurysm. Antibiotics-coated grafts were not available at our hospital, which we acknowledged by washing the operative area with amikacin after the graft placement. The omental flap was mobilized and wrapped around the graft to isolate it from the surrounding tissues, which were the main suspects for the infection source. Silver-coated and rifampin-bonded grafts have shown a lower infection rate, but the published data is scarce [6].

## 4 Conclusion

Here we report peculiar clinical picture of an aortic aneurysm transformed into a mycotic aortic aneurysm after gastroenteritis. That aneurysm quickly grew by 3 cm in two weeks and reached 10 cm in size without rupturing. This emphasizes the importance of careful monitoring of aneurysms and the necessity of an early intervention in appropriate manner, especially if there is any infectious condition in the body.

## Ethical approval

Not required.

## Funding

No funding was required.

## Author contribution

All authors contributed to this work.

GB: collecting references, original draft writing and editing.

R.I.: reviewing and supervision.

H.K. and H.K.: final reviewing and supervision.

## Guarantor

Rana Issa is the guarantor of this work.

## Registration of research studies

Not required.

## Generative AI in scientific writing

None.

## Patient's consent

Written informed consent was obtained from the patient for publication of this case report and any accompanying images. A copy of the written consent is available for review by the Editor-in-Chief of this journal upon request.

## Declaration of competing interest

None.
